# Supplementary material for: Assessing Healthcare Workers’ Knowledge and Their Confidence in the Diagnosis and Management of Human Monkeypox: A Cross-Sectional Study in a Middle Eastern Country
Source: Healthcare (Basel). 2022 Sep 8;10(9):1722. doi: 10.3390/healthcare10091722 (PMC9498667; doi:10.3390/healthcare10091722)
Supplement: Supplementary file 1 [file healthcare-10-01722-s001.zip › Consent_Form.pdf]

Consent form

Knowledge of Healthcare Workers in Jordan Regarding Monkeypox and their Confidence in Diagnosis and Management

This project aims to assess the knowledge of Jordanian healthcare workers regarding monkeypox, and their attitude towards possible explanations of virus emergence.

The potential benefits of this research include highlighting the gaps of knowledge and assessment of confidence levels to diagnose and manage the emergent monkeypox disease.

The information provided by you in this questionnaire will be used for research purposes. It will not be used in a manner which would allow identification of your individual responses.

The survey is estimated to take about 5-10 minutes to be completed.

Principal investigators: Malik Sallam, Faris Bakri and Azmi Mahafzah

Contact details: Dr. Khaled Al-Salahat, resident, phone: +96279797322093

Thank you very much for agreeing to take part in this survey.

"Do you agree to participate in this study?

☐ Yes

☐ No
