# Supplementary material for: Assessing Healthcare Workers’ Knowledge and Their Confidence in the Diagnosis and Management of Human Monkeypox: A Cross-Sectional Study in a Middle Eastern Country
Source: Healthcare (Basel). 2022 Sep 8;10(9):1722. doi: 10.3390/healthcare10091722 (PMC9498667; doi:10.3390/healthcare10091722)
Supplement: Supplementary file 1 [file healthcare-10-01722-s001.zip › Ethical-Permission-Confidential-No-to-be-published.pdf]

كلية الطب

SCHOOL OF MEDICINE

الجامعة الأردنية

THE UNIVERSITY OF JORDAN

الرقم: 2/544/2022/67

التاريخ: 2022/5/31

السادة رؤساء الأقسام المعنية

الموضوع: تنسيبات لجنة البحث العلمي (13)- بخصوص الأبحاث (غير) المدعومة

تحية طيبة وبعد،،

فقد ناقشت لجنة البحث العلمي في الكلية في جلستها رقم (2022-2021/13) المنعقدة بتاريخ 2022/5/24، عدداً من الأبحاث (غير) المدعومة، وقد نسبت اللجنة بالموافقة على تسجيل الأبحاث كالتالي:

قسم الجراحة الخاصة

1- البحث المقدم من د. طارق محافظة بعنوان:

Is routine preoperative coagulation study necessary? An audit conducted at a tertiary teaching hospital

1- البحث المقدم من د. المعتز غرايبة بعنوان:

Oil Based Solid Ascorbic Acid as a Novel Formulation For Corneal Epithelial Regeneration

شريطة أخذ موافقة IRB؛ حيث إنها دراسة تداخلية.

قسم الجراحة العامة

1- البحث المقدم من د. محمد الرشدان بعنوان:

Mesh fixation techniques in TAPP : Fibrin glue vs. tackers , a randomized controlled trial

قسم الأمراض الباطنية

1- البحث المقدم من د. حسام الحوري بعنوان:

Comparison of Outcome of American thyroid association (ATA) Low-risk Differentiated Thyroid Cancer (DTC) Treated with low vs high administered Iodine-131 Activity

2- البحث المقدم من د. خالد عويدات بعنوان:

Comparing Positional and Non-Positional Obstructive Sleep Apnea patients among Jordanian Population

3- البحث المقدم من د. مروان العدوان بعنوان:

Patient satisfaction in SLE treatment: A single center study

قسم طب الأطفال

1- البحث المقدم من أ.د أميرة المصري بعنوان:

Genetic etiologies underlying developmental epileptic encephalopathies in children

قسم النسائية والتوليد

1- البحث المقدم من د. ناصر الحسبان بعنوان:

Are females who consume heavily processed meat and hydrogenated fats more prone to laparoscopy-diagnosed endometriosis than others?

2- البحث المقدم من د. عقبة القرعان بعنوان:

Histopathologic findings among patients who underwent hysterectomy, a case-series.

هاتف: ٥٣٥٥٠٠٠ (٦-٩٦٢) فرعي: ٢٣٤٠٠ فاكس: ٥٣٠٠٨٢٠ (٦-٩٦٢) داخلي- ٢٣٥١١ عمان ١١٩٤٢ الأردن
Tel.: (962-6) 5355000 Ext.: 23400 Fax: (962-6) 5300820 Ext: 23511 Amman 11942 Jordan
http://medicine.ju.edu.jo E-mail: deanmed@ju.edu.jo

قسم علم الأمراض والأحياء الدقيقة والطب الشرعي

1- الأبحاث المقدمة من د. مالك سلام بعنوان:

1- Levels of knowledge of human monkeypox viral infection among Jordanian healthcare workers and its relation to conspiracy explanations regarding emerging virus infections.
2- Levels of knowledge of human monkeypox viral infection among the general public in Jordan its relation to conspiracy explanations regarding emerging virus infections.
3- Levels of knowledge of human monkeypox viral infection among the students in health faculties/schools in Jordan its relation to conspiracy explanations regarding emerging virus infections.

وتفضلوا بقبول فائق الاحترام،،،

عميد كلية الطب

الأستاذ الدكتور ياسر ريان

- ملاحظة: يرجى من الأقسام المذكورة تزويد الباحث الرئيس بنسخة من هذه الموافقة، ليقوم بتحميل ما يلي على موقع الأبحاث العلمية في المستشفى:
http://apphosp.ju.edu.jo/scientific_research

(نسخة من هذا الكتاب & نموذج البحث معباً حسب الأصول (في حال احتاج لموافقة لجنة IRB بالمستشفى) & أي مرفقات للبحث، كالاستبيان، وغيره).

- نسخة: الباحثين.
- نسخة: أمينة سر اللجنة.

هاتف: ٥٣٥٥٠٠٠ (٦-٩٦٢) فرعي: ٢٣٤٠٠ فاكس: ٥٣٠٠٨٢٠ (٦-٩٦٢) داخلي- ٢٣٥١١ عمان ١١٩٤٢ الأردن
Tel.: (962-6) 5355000 Ext.: 23400 Fax: (962-6) 5300820 Ext: 23511 Amman 11942 Jordan
http://medicine.ju.edu.jo E-mail: deanmed@ju.edu.jo
