# Supplementary material for: Assessing Healthcare Workers’ Knowledge and Their Confidence in the Diagnosis and Management of Human Monkeypox: A Cross-Sectional Study in a Middle Eastern Country
Source: Healthcare (Basel). 2022 Sep 8;10(9):1722. doi: 10.3390/healthcare10091722 (PMC9498667; doi:10.3390/healthcare10091722)
Supplement: Supplementary file 1 [file healthcare-10-01722-s001.zip › Supplementary.pdf]

Supplementary

Consent form and questionnaire translated to English

Knowledge of Healthcare Workers in Jordan Regarding Monkeypox and their Confidence in Diagnosis and Management

This project aims to assess the knowledge of Jordanian healthcare workers regarding monkeypox, and their attitude towards possible explanations of virus emergence.

The potential benefits of this research include highlighting the gaps of knowledge and assessment of confidence levels to diagnose and manage the emergent monkeypox disease.

The information provided by you in this questionnaire will be used for research purposes. It will not be used in a manner which would allow identification of your individual responses.

The survey is estimated to take about 5-10 minutes to be completed.

Principal investigators: Malik Sallam, Faris Bakri and Azmi Mahafzah

Contact details: Dr. Khaled Al-Salahat, resident, phone: +96279797322093

Thank you very much for agreeing to take part in this survey.

"Do you agree to participate in this study?

Yes

No

---

1. Age

………………….

2. Sex

Male

Female

3. Educational level (the highest level of formal education completed):

Undergraduate (diploma or BSc degree)

Postgraduate (MSc, PhD or its equivalent)

4. Place of residence

The Capital (Amman)

Outside the Capital

5. Occupational category

Physician

Dentist

Pharmacist

Nurse

Medical technician (laboratory, radiology, rehabilitation, or anesthesia technician)

Human monkeypox knowledge

Please answer the following questions with yes, no, or I do not know based on your current knowledge of monkeypox

6. Monkeypox is prevalent in the Middle East

Yes

No

I do not know

7. Monkeypox is prevalent in Western and Central Africa

Yes

No

I do not know

8. There is an outbreak of human monkeypox in the world

Yes

No

I do not know

9. Monkeypox is caused by a virus

Yes

No

I do not know

10. Human-to-human transmission of monkeypox occurs easily

    Yes

    No

    I do not know

11. Monkeypox and smallpox have similar signs and symptoms

    Yes

    No

    I do not know

12. Skin rash is one of the signs or symptoms of human monkeypox

    Yes

    No

    I do not know

13. Pustule is one of the signs or symptoms of human monkeypox

    Yes

    No

    I do not know

14. Antibiotics are used to treat human monkeypox

    Yes

    No

    I do not know

15. Diarrhea is one of the signs or symptoms of human monkeypox

    Yes

    No

    I do not know

16. Vaccination is available to prevent human monkeypox

    Yes

    No

    I do not know

Confidence in the diagnosis and management of monkeypox

17. Are you confident to diagnose monkeypox cases based on your current knowledge and skills?

Yes

No

18. Are you confident to diagnose monkeypox cases based on the ability of your current facility to do diagnostic test?

Yes

No

19. Are you confident to manage monkeypox cases, if any, based on your current knowledge and skills?

Yes

No

The attitude towards the emergence of viruses and the global/local response to outbreaks/epidemics (Please answer each question based on your opinion)

**Item**	Strongly Disagree	Disagree	Somewhat Disagree	Neutral/ No opinion	Somewhat Agree	Agree	Strongly Agree
20. Monkeypox spread worldwide due to the role of male homosexuals							
21. I am skeptical about the official explanation regarding the cause of virus emergence							
22. I do not trust the information about the viruses from scientific experts							
23. Most viruses are man-made							
24. The spread of viruses is a deliberate attempt to reduce the size of the global population							
25. The spread of viruses is a deliberate attempt by governments to gain political control							

26. The spread of viruses is a deliberate attempt by global companies to take control							
27. Lockdowns in response to emerging infection are aimed for mass surveillance and to control every aspect of our lives							
28. Lockdowns in response to emerging infection are aimed for mass surveillance and to destabilize the economy for financial gain							
29. Lockdown is a way to terrify, isolate, and demoralize a society as a whole in order to reshape society to fit specific interests							
30. Viruses are biological weapons manufactured by the superpowers to take global control							
31. Coronavirus was a plot by globalists to destroy religion by banning gatherings							
32. The mainstream media is deliberately feeding us misinformation about the virus and lockdown							

Thank you very much for participating in the survey
